# Synthesis and characterization of edible films using high methoxyl pectin extracted from orange peel

**DOI:** 10.1038/s41598-026-43924-8

**Published:** 2026-04-02

**Authors:** Hassan R. S. Abdellatif, Marwa M. Helmy, Heba G. R. Younis

**Affiliations:** 1 Agricultural Engineering Department, Faculty of Agriculture, Cairo University, Giza, 12613 Egypt; 2 Food Technology Research Institute, Agricultural Research Center, Giza, Egypt

**Keywords:** Antioxidant activity, Biodegradable test, Orange peel, Acid extraction, Mechanical properties, High methoxyl pectin, Biotechnology, Chemistry, Engineering, Environmental sciences

## Abstract

This study aimed to examine the effectiveness of the extraction of pectin from orange peel waste using hydrochloric acid and citric acid and how they affect the quantity and quality of pectin, as well as the potential uses of the extracted pectin in edible film formulations. Both extraction procedures were used to extract orange peel samples at 80 °C for one hour. The results showed that using hydrochloric acid to extract pectin (HP) achieved slightly better results (15.15%) than using citric acid, CAP (14.44%). Chemical analysis showed that the two methods were quite different: the pectin from hydrochloric acid had more moisture (3.00%) with a higher equivalent weight of 806.45 mg/mol, while the pectin from citric acid had more ash (1.952%) and a lower equivalent weight of 769.23 mg/mol. Both extraction techniques maintained critical functional properties, and esterification values remained within market standards (51.85%). The extracted pectin was effectively used to make edible films, indicating its potential for other valuable applications. The edible films were characterized by mechanical properties, WVP, optical properties, SEM, FTIR, XRD, DPPH, and biodegradability. The citric acid edible films had the highest DPPH and tensile strength, the lowest elongation and WVP, and the highest degradability in soil. So, the citric acid extraction procedure was demonstrated to be environmentally friendly and lower environmental impact. This study provides valuable insights into producing pectin in an environmentally friendly manner for the food and packaging sectors.

**Supplementary Information:**

The online version contains supplementary material available at 10.1038/s41598-026-43924-8.

## Introduction

The citrus juice industry in Egypt generates substantial orange peel waste, estimated at 1.7–2.2 million metric tons (MMT) annually, as peels comprise 40–50% of fruit mass from the country’s ~ 4.35 MMT citrus production^1^. These peels offer significant valorization potential for various applications. They are abundant in bioactive compounds, including polyphenols, flavonoids, carotenoids, essential oils (such as limonene), dietary fibers, and minerals, exhibiting potent antioxidant, antimicrobial, and anti-inflammatory properties^2^. Citrus fruits, especially oranges, are among the most widely grown fruits in the world. Citrus fruits are grown in large quantities because they have strong sensory qualities (taste, smell, and flavor) and health benefits^3,4^. The global citrus production in 2023 reached approximately 160 million tonnes annually. This results in substantial waste, with peels comprising about half of the fruit, totaling around 80 million tonnes^5^. Egypt produces approximately 4.35 MMT of citrus annually (2023), predominantly oranges (~ 80%). Egypt ranks among the top global producers and exporters of citrus, led by oranges, with fresh orange exports reaching 1.95 million metric tons (MMT) in marketing year (MY) 2024/2025^5^. Approximately one-third of the food produced, almost 1.3 billion metric tons, is squandered each year during harvesting, preservation, transportation, marketing, and consumption; fruits and vegetables constitute approximately 45% of total food waste^6^.

In 2020, about 14% of food intended for human consumption was lost globally before market entry, resulting in an annual loss of $400 billion^7^. Orange peels are a byproduct of the citrus juice industry in Egypt, where hundreds of tons of oranges are processed annually to produce juice for both domestic and international markets. This has led to extensive research into the economic potential of citrus processing waste to find other uses for this byproduct that will increase market demand and raise its overall value in the economy^4^. The peel and pulp (the portion separated from the liquid) constitute the orange residue. Cellulose, pectin (galacturonic acid), hemicellulose, lignin, chlorophyll pigments, and other low molecular weight substances compose the majority of its composition. These components are mostly composed of pectin, cellulose, hemicelluloses, lipids, some nitrogen compounds, and nearly 3% ash content^8^. The primary type of polysaccharide present in orange residual cell walls is pectic compounds^8^.

There are several ways to utilize citrus processing waste, including composting, feeding animals, and producing organic fertilizers. Additionally, producing biogas and extracting valuable components such as pectin, bioactive materials, and essential oils from this waste can make it more beneficial for food packaging. Therefore, emphasizing the diverse potential applications of citrus processing waste is crucial for enhancing its utilization and supporting the sustainability of the citrus sector^4,9^. Pectin, a complex heterogeneous polysaccharide and significant hydrocolloid, is widely utilized in edible films, food, pharmaceuticals, and cosmetics due to its non-toxicity, low cost, ready availability, and excellent gelling, thickening, and stabilizing properties^6,10–13^. Pectin can be derived from citrus peels, and it is necessary to identify more efficient extraction methods and optimal applications for commercial use^14,15^. Citrus pectin is a byproduct of the citrus juice industry and is presently the primary commercial source of pectins^6,16^. Pectin can be obtained from fresh dragonfruit peels through subcritical water extraction. It may function as an edible film with superior thermal stability, improved moisture barrier properties, lighter coloration, and greater transparency than pectin films extracted with acid^10^. The pectin content is roughly 10–20 g per 100 g of dried passion fruit peel, indicating its significant potential as a pectin source^16^. Plastic materials, widely used in food packaging, are primarily non-renewable and non-degradable petroleum-based products. The disposal of plastic films made by the chemical industry could harm the environment as hazardous chemicals, gases, and toxins leach from landfills during disposal, causing severe soil and groundwater contamination that threatens plants, animals, and human health^17^. Biodegradable packaging and edible films derived from renewable resources provide sustainable solutions to the global plastic pollution crisis^18^; So there has been a surge in interest in eco-friendly alternatives made from natural resources or bio-based polymer films (proteins, lipids, chickpea starch, chitosan, Potato starch and pectin (PE); these materials are renewable, non-toxic, and ecologically friendly and produce biodegradable films that can extend the shelf life of agricultural products during storage^19–25^. This study primarily aims to valorize orange peels by systematically comparing two extraction methods: mineral acid extraction with hydrochloric acid (HCl) and organic acid extraction with citric acid (C_6_H_8_O_7_). This work integrates both phases to comprehensively evaluate how the extraction medium influences pectin yield, structural properties, and functional utility. Specifically, we demonstrate the novel synergistic role of citric acid as both a sustainable solvent and functional crosslinker. By linking extraction efficiency to the physicochemical, mechanical, and morphological properties of the resulting edible films, this research bridges waste management and materials science, thereby validating a comprehensive circular-economy approach for biodegradable food packaging.

## Materials and methods

### Materials

Citric acid monohydrate (99.5%), Ethyl alcohol 96% (Ethanol) were purchased from Piochem, Hydrochloric acid (30–34%) and Acetic acid (96%) were obtained from El Nasr Pharmaceutical Chemicals Co. (Egypt). Glycerol was purchased from Diachem Chemicals (Egypt). The chemicals employed in the extraction procedure were of analytical purity. Fresh orange peel waste was collected, cleaned, and subjected to direct solar drying until a constant weight was achieved, ensuring a stable moisture content of approximately 10%. Then desiccated, ground, and sealed in an airtight at ambient temperature until needed for extraction activities.

### Pectin extraction process

Pectin was extracted from the orange peel powder using a hot acidic extraction method (HCl and citric acid). The extraction was performed using a solid-to-liquid ratio of 1:20 (w/v). The mixture was maintained at 90 °C for 1 h with continuous stirring. This duration was selected based on preliminary optimization trials, which demonstrated that 60 min provided the maximum yield while preventing the hydrolytic degradation of the polysaccharides; beyond this period, no significant increase in recovery was observed, as described by El Fihry et al.^26^, with some modifications. The resulting material was dried at 50 °C to remove moisture until a constant weight was achieved. It was subsequently ground and stored in airtight plastic bags for analysis.

### Characterization of pectin extracted

Standard titrimetric methods were used to determine pectin yield, moisture content, ash content, equivalent weight (EW), methoxyl content, total anhydrouronic acid (AUA), and degree of esterification (DE) (see *Supplementary* for comprehensive protocols and equations). Phenolphthalein or phenol red indicators were used in all titrations with 0.1 N NaOH.

### Preparation of edible films

Pectin edible films were prepared using the casting method, as described by Younis et al.^27^. Solutions containing 0.5, 1.0, 1.5, and 2.0% pectin extracted by the two methods were dissolved in distilled water containing 0.5% acetic acid. The mixture was exhaustively agitated at 90 °C until a clear solution was obtained. After the resultant solution cooled to 30 °C, glycerol (0.5%, w/w) was added to the solution. The solution was then poured into molds and dried in an oven at 40 °C for 24 h. Finally, the dried film was peeled off and stored at room temperature.

### Characterization of edible films

#### Thickness and water vapor permeability

A digital micrometer (Mitutoyo, Model MDC-25 S, 0.001 mm, USA) was used to determine the thickness. The water vapor transmission rate (WVTR) [g/(s.m^2^)] and water vapor permeability (WVP)[g.mm/mm^2^.day.mmHg] of the films were determined gravimetrically using the ASTM E96/E96M-16 Method. Before characterization, all films were conditioned at 50% RH and 25 °C for 48 h to achieve moisture equilibrium. Equations 1 and 2 were used to determine the WVTR and WVP.

1 $${WVTR}=\frac{\varDelta m}{\varDelta t*A}$$

2 $$WVP=WVTR*\frac{{L}}{\varDelta {R}{H}}$$

Where A is the film’s surface area (m^2^), L is the thickness (mm), ΔRH is the relative humidity difference, and ∆m/∆t is the moisture gain weight per time (g/s).

#### Mechanical properties

The mechanical properties were determined using a Texture Analyzer (Brookfield CT-3, USA) at a crosshead speed of 50 mm/min and an initial gauge length of 50 mm, according to the ASTM D882 standard. The samples were clamped between pneumatic grips, and a force of 10–50 KN was applied.

#### Transparency and optical properties

The transparency of the film was estimated using a spectrophotometer (Linshang, LS108, China) with a visible light wavelength of 550 nm. The color parameters L (luminosity), a (- greenness; + redness), and b (- blueness; + yellowness), as well as total color difference (ΔE) for the different films were assessed by the 3nh Portable precision colorimeter (NR10QC, China).

#### Biodegradability test

The biodegradation test in soil for the different pectin films was analyzed by simulating the degradation conditions in a natural soil environment^28,29^, with some modifications. A soil burial experiment was conducted to assess the degradation of the different films. The films were cut to 20 mm × 20 mm and buried in soil at a depth of 5 cm to assess degradation over 30 days.

### Radical DPPH scavenging activity of pectin powder and pectin films

The free radical scavenging capacity of the extracts was determined using the stable DPPH (2,2-diphenyl-1-picrylhydrazyl) method, as described by Bello and Peresin^30^ with some modification. The final concentration of DPPH was 200 µM, and the final reaction volume was 3.0 mL. The absorbance was measured at 517 nm against a blank of pure methanol after 60 min of incubation in dark conditions.

### Structural characterization of pectin powder and pectin films

The extracted pectin and different pectin films were analyzed using FT-IR Spectrometer (Invenio, Bruker 65, German), the wavenumber region recorded for the analysis from 4000 –400 cm^− 1^. X-ray Diffraction was used to determine the diffraction patterns (X’Pert Pro PW1730, PANalytical, Netherlands) with a secondary monochromator, using Cu-radiation (λ = 1.542 Å) at 45 kV, 35 mA, and a scanning speed of 0.04^°^/sec, at diffraction angles 2θ from 2° to 60°. The different pectin films were analyzed for morphological features using a scanning electron microscope SEM (Quanta 250 FEG, Netherlands). The complete methodology for valorizing orange peel waste, pectin extraction through HCl/CAP, pectin characterization, edible film Synthesis, and circular packaging testing is summarized schematically in Fig. S1 (Supplementary).

### Statistical analysis

All tests were performed in triplicate, and the data were statistically analyzed using one-way ANOVA analysis (SPSS 25.0, USA), with Tukey’s test at *p* < 0.05.

## Results and discussion

### Characterization of extracted pectin

#### Physicochemical Properties of Pectin

The two techniques of extracting pectin showed considerable differences in yield and chemical characteristics. The results indicate that hydrochloric acid extraction (HP) yielded 15.15% pectin, slightly higher than the 14.44% obtained from citric acid extraction (CAP). This difference can be attributed to the stronger acidic nature of HCl, which more effectively breaks down the cell wall structure of orange peels, releasing a greater amount of pectin. Additionally, HCl facilitates network stabilization and the formation of extended pectin chains by neutralizing the negative charges on the carboxyl groups. This reduction in electrostatic repulsion allows for a more cohesive arrangement of the polymer, thereby enhancing the overall extraction efficiency. These results suggest that hydrochloric acid is slightly more effective than citric acid in stabilizing the pectin matrix during the extraction process^31,32^.

Control of the moisture content in pectin is necessary for storage. The low moisture content of the extracted pectin (3% for HP, 2.5% for CAP) is within the acceptable range for high-quality pectin, as specified by Lan et al.^33^. Maintaining the stability of pectin is crucial for preserving its advantageous properties in edible films and other applications, where a low moisture content is necessary to maintain pectin’s stability during storage. This is because it inhibits the growth of microorganisms, particularly fungi, which can degrade the substance over time. The slight numerical difference in moisture content, where HCl extracted pectin (HP) exhibited 3% compared to 2.5% for citric acid extracted pectin (CAP) may be attributed to the high concentration of hydronium ions in the HCl medium. These ions can increase the hygroscopicity of the pectin network, leading to a marginally higher retention of bound water during the final drying phase. However, both values demonstrate a highly stable, low-moisture product suitable for active packaging applications. On the other hand, the ash content gave different findings. The CAP produced a greater ash percentage (1.952%), which was similar to that of CP (1.902%). HCl extraction, on the other hand, gave a much lower ash concentration (0.95%), which is crucial for the gelling and film-forming properties of pectin. These values are well below the upper limit of ash content considered acceptable for good-quality pectin, which is 10%. This contrast indicates that a higher retention of mineral elements from the orange peel matrix may take place during citric acid extraction, thus enhancing the purity and low mineral residue of the obtained pectin. High ash content can interfere with pectin properties, making the pectin less suitable in food and pharmaceutical applications^34,35^. Equivalent weight is a crucial characteristic of pectin, influencing its ability to form gels. The equivalent weight of CP was 714.286 mg/mol, while the equivalent weights of HP and CAP were found to be 806.45 and 769.23 mg/mol, respectively. The International Pectin Producers Association suggests a minimum equivalent weight of 400 mg/mol^3^. The equivalent weights in this study were higher than those reported for jackfruit peel and core (475.74 and 460.63 mg/mol, respectively) by Ahmmed et al.^36^.

**Table 1:** Physicochemical Properties of Pectin by citric acid and HCl.

Parameters	Commercial pectin (CP)	Citric acid extraction pectin (CAP)	HCl extraction pectin (HP)
Moisture content, %	2.04 ± 0.05^a^	2.50 ± 0.09^b^	3.00 ± 0.10^c^
Ash content,%	1.902 ± 0.02^b^	1.952 ± 0.05^b^	0.950 ± 0.07^a^
Equivalent weight	714.29 ± 2.18^a^	769.23 ± 2.50^b^	806.45 ± 1.04^c^
Methoxyl content,%	4.34 ± 0.13^a^	3.84 ± 0.36^a^	4.03 ± 0.13^a^
Degree of Esterification,%	51.85 ± 0.77^b^	50.15 ± 1.10^a^	50.98 ± 0.90^ab^
Anhydrouronic acid (AUA),%	47.52 ± 1.59^a^	44.35 ± 1.21^a^	44.88 ± 1.41^a^

Means ± SD (*n* = 3) within the same row followed by different superscript letters (a, b, c) differ significantly (one-way ANOVA followed by Tukey’s LSD test, SPSS 25.0, *p* < 0.05).

The high equivalent weight of pectin would indicate a stronger gel-forming ability, which the amount of free acid present may influence in the pectin samples^37^. Increased equivalent weight values indicate more uniform molecular weight distributions and potentially varying degrees of polymerization, which may affect the physical and functional properties of the extracted pectin. This result confirms that the extraction conditions significantly influence the structure and function of pectin. Also, the equivalent weight varies depending on the source of pectin, the extraction method, and the type of acid used, with a higher equivalent weight corresponding to better gel-forming quality^38^. Methoxyl content is another critical aspect of pectin analysis, as it provides information on the degree of esterification of the polygalacturonic acid chains in pectin. The methoxyl content of extracted pectin was investigated using citric acid and HCl from the same source. The results show that the methoxyl contents of CP were 4.34%, while the values for CAP and HP were 3.84% and 4.03%, respectively. The relatively low methoxyl content may be attributed to the type of acid used and the moderate extraction power of the methods, which depolymerized the galacturonan chains into shorter polygalacturonic acid chains. These findings are in line with various studies, notably those by Salma et al.^35^ observed a methoxyl concentration of 1.56% in lemon peel pectin, but Islam et al.^39^ mentioned a methoxyl content ranging from 2.98 to 4.34% in dragon fruit pectin. Depending on the pectin source and extraction technique, Aina et al.^40^ found a broad range of methoxyl concentrations ranging from 0.2 to 12%. Pectin with less than 7% methoxyl content is categorised as low methoxyl pectin (LMP), which forms gels with lower sugar concentrations or without sugar^41^. The degree of esterification (DE) is considered an essential tool for categorizing pectin according to the proportion of carboxyl groups esterified with methanol and evaluating pectin’s applicability in the food industry, as it influences gelation, stability, and emulsification^42^. The results showed that the DE values for CP, CAP, and HP were 51.58%, 50.15%, and 50.98%, respectively, indicating that the extracted citrus pectin samples are high methoxyl pectin (HMP). Pectin is classified as high methoxyl pectin (HMP; DE > 50%) or low methoxyl pectin (LMP; DE < 50%) according to its degree of esterification. LMP can form gels when combined with divalent cations such as Ca²⁺, making it valuable for applications in both the food and pharmaceutical industries. In contrast, HMP is mainly used to produce jams, jellies, and confectionery products^6,18^. High methoxyl pectin (HMP) exhibits effective emulsifying properties, particularly in high internal phase emulsions (HIPEs) and protein mixtures, while also forming strong, smooth, edible films with excellent mechanical properties and low water vapor permeability-often enhanced by plasticizers or lipids for food packaging applications such as fruit preservation^18,43^. Anhydrouronic acid (AUA) is a key parameter for assessing the purity of extracted pectin, where a higher galacturonic acid and lower ash content indicate greater purity. The results showed that the AUA content obtained from CP was 47.52%, while the values for CAP and HP were 44.35% and 44.88%, respectively. AUA content below 60% may indicate the presence of impurities such as protein, starch, and sugars in precipitated pectin^44^. However, these values are comparable to those reported for banana peel pectin 53.60% and apple peel pectin 62.82%^45,46^.

#### FT-IR of extracted pectin

**Fig. 1:**
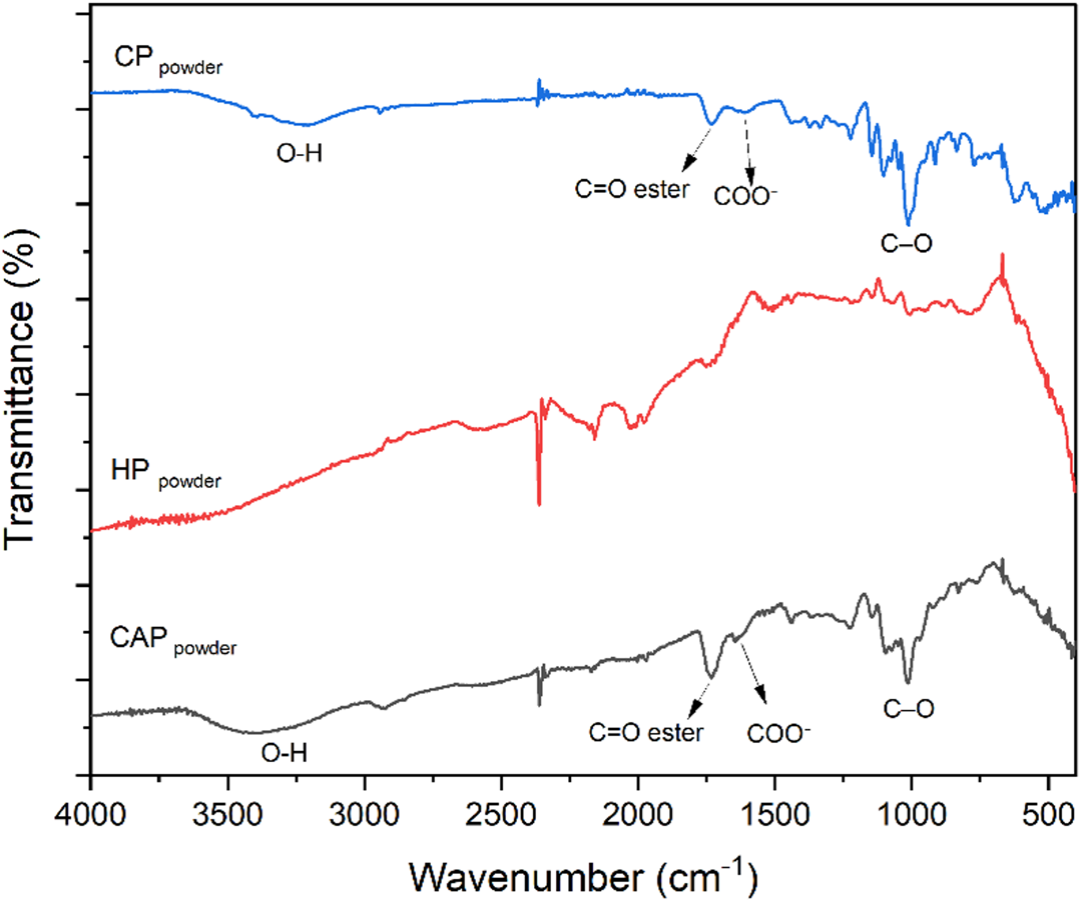
FTIR spectra of pectin extracted (CAP, HP) and CP powder.

The structure of pectin HP, CAP, and CP was analyzed using FT-IR spectroscopy as shown in Fig. 1. The absorbance bands exhibited noticeable variations and shifts. The peak observed at approximately 3323 cm^− 1^ is attributed to the O-H stretching, which is less intense than that of pectin obtained through the HCl extraction method, likely due to the intramolecular hydrogen bonding of the galacturonic acid backbone, and is reflected in the moisture content value^47,48^. The peaks at 1736 cm^− 1^ in the FTIR spectra demonstrate the existence of carboxylic groups in the pectin, while the spectral region below 1500 cm^[− 148–50^, known as the “fingerprint” region of pectin, shows variations in intensity and displacement of the vibration of the 1226 cm^− 1^, vibration corresponding to the –CH_3_CO group, among the different samples. The peak observed at 1014 cm^− 1^ is attributed to C–O stretching from glycosidic bonds^47^. The absorption 823 cm^− 1^ was assigned to the C–H deformation of the α-glycosidic linkages, characteristic of the polygalacturonic acid backbone, and 930 cm^− 1^ is associated with the C–O–C vibration of glycosidic linkages in neutral sugar side chains^51,52^. The 1740/1620 cm⁻¹ ratio indicates borderline HMP characteristics (DE ~ 50%) consistent with titration. The difference in DE values obtained in Table 1 and FTIR measurements is attributed to the presence of impurity residues in pectin^53^.

### Characterization of edible films

#### Physical properties of edible films

**Fig. 2:**
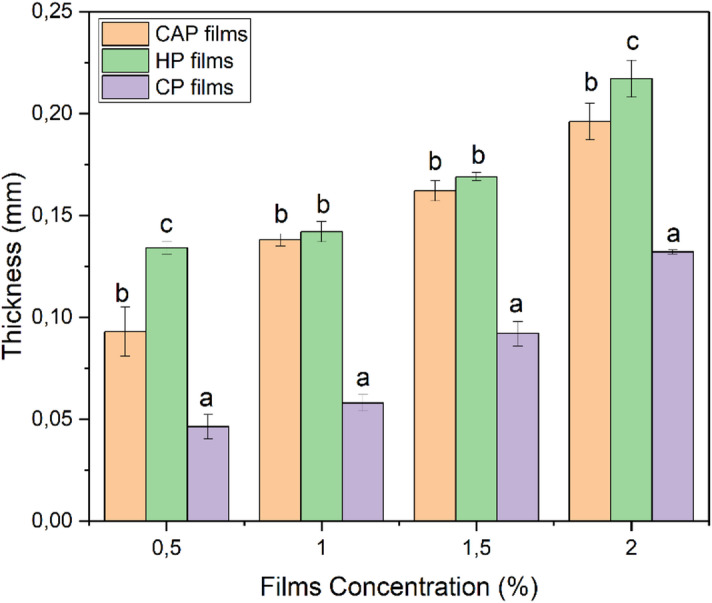
Thickness of edible films (CAP, HP, and CP) at different pectin concentrations. Different bars indicate significant differences (one-way ANOVA followed by Tukey’s LSD test, SPSS 25.0, *p* < 0.05).

The performance of edible films relies on their structural compactness and mechanical strength, both of which are enhanced by film thickness^54^. The values for thickness varied from 0.0463 to 0.271 mm, as shown in Fig. 2. The CP films were found to have reduced thickness values, which are consistent with Yang et al.^54^ findings. HP films, on the other hand, had the highest thickness readings. At varying concentrations, no variations between CP and CAP films were found.

Additionally, there were no significant differences among the films at the same concentration. However, thickness increased significantly with increasing concentration for all film types.

**Fig. 3:**
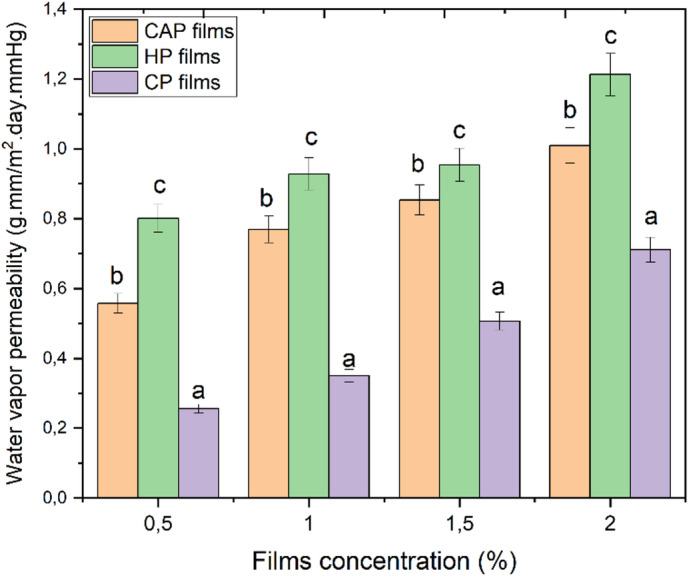
Water vapor permeability at different concentrations of edible films. Different bars indicate significant differences (one-way ANOVA followed by Tukey’s LSD test, SPSS 25.0, *p* < 0.05).

Water vapor permeability WVP is one of the critical properties of films for food packaging applications, as it limits water exchange between the product and the surrounding environment, thereby preventing food spoilage^54,55^. Figure 3 presents WVP values of different films. CP films exhibited the lowest WVP values (*P* ≤ 0.05) at all concentrations compared to films prepared from CAP and HP. At the lowest concentration, 0.5%, the WVP values recorded for CP, CAP, and HP films were 0.255, 0.557, and 0.801 g.mm/mm^2^.day.mmHg respectively.

As the concentrations increased, WVP values also increased. At the highest concentration (2%), WVPs were 0.711, 1.01, and 1.213 g.mm/mm^2^.day.mmHg, respectively, for CP, CAP, and HP films as shown in Fig. 3. It was observed that films prepared using acid in extracted pectin exhibited higher values of WVP. This phenomenon might be ascribed to the impact of hydrophilic groups, as heightened acidity during extraction may compromise the pectin macromolecular structure, thereby revealing a greater number of hydrophilic functional groups (e.g., carboxyl groups). These groups facilitate water absorption and enhance water transport; the increased presence of these groups likely facilitated the transport of water molecules through the matrix via a solubility diffusion mechanism. Furthermore, the hygroscopic nature of the extracted pectin may have led to a more open swollen structure at higher concentrations, thereby increasing the free volume and facilitating water vapor transmission, which explains the increase in WVP values ​​with higher extracted pectin concentration^51^.

#### Mechanical properties of edible films

**Fig. 4:**
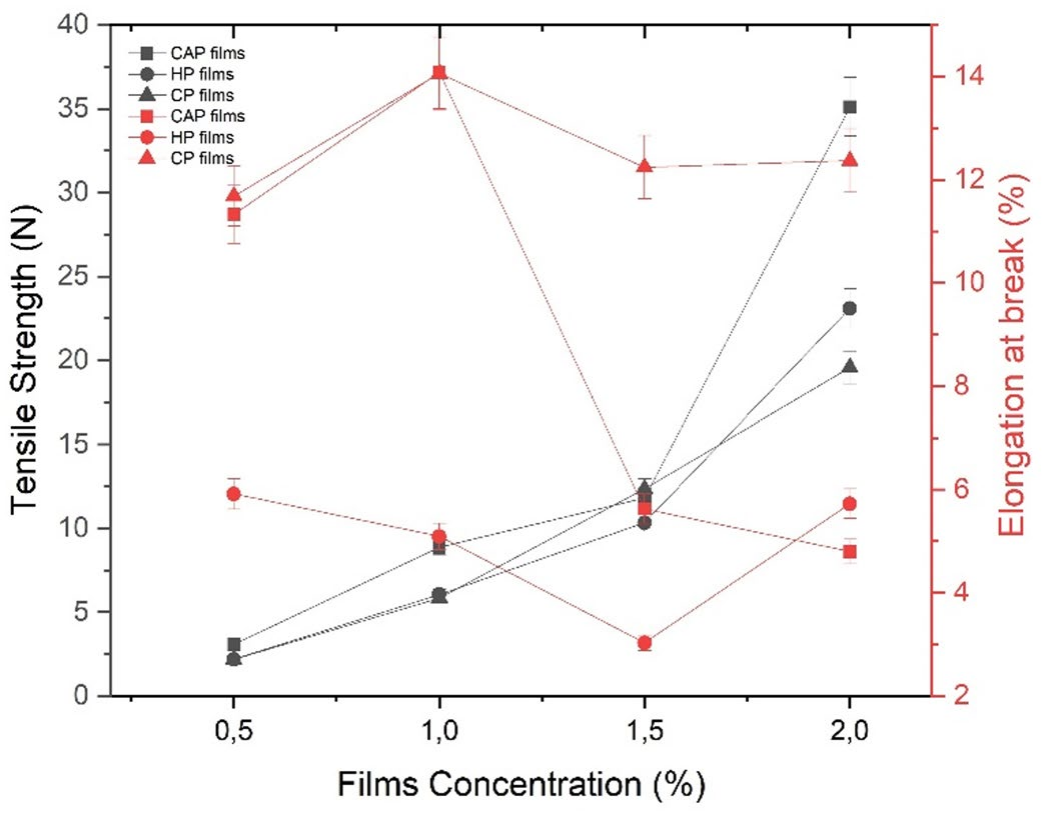
Tensile strength and Elongation at different concentrations of edible films. Different bars indicate significant differences (one-way ANOVA followed by Tukey’s LSD test, SPSS 25.0, *p* < 0.05).

To ensure that the edible films retain structural integrity, packaging films must possess sufficient mechanical strength^56^. The mechanical characteristics, TS and EB, of the various films are shown in Fig. 4. Mechanical resistance is their tensile strength (TS), which is based on the cohesive forces among the polymer chains. Another measure of a film’s flexibility is its elongation at break (EB), which implies its capacity to stretch before rupture. The structural characteristics of these attributes indicate that films with high tensile strength demonstrate slight elongation at break. Consequently, both parameters must be examined together^57^. These characteristics are crucial because they directly influence film performance and are correlated with the distribution and density of intermolecular and intramolecular interactions within the polymer matrix^54^. The links within these networks affect TS, while molecular interactions dictate the flexibility of the pectin film. High tensile strength in food packaging is crucial for safeguarding food during handling, transit, and marketing.

As illustrated in Fig. 4, the CAP film exhibited the highest TS value (35.11 N) at 2% concentration. In contrast, CP film had the lowest TS value (19.57 N), and HP film recorded TS value (23.10 N) at the same concentration. CAP films exhibited the highest TS values, 3.07 N and 8.86 N at 0.5% and 1.0% concentrations, respectively, whereas at the same concentrations, HP films and CP films showed no significant differences. At a concentration of 1.5%, the CP film exhibited the highest TS value, 12.34 N, followed by the CAP film, 11.8 N, and the HP film, 10.33 N. The elongation at break (EB) of HP films was consistently the lowest across concentrations, while CAP and CP films showed no significant differences at 0.5% and 1.0% concentrations. However, at 1.5% and 2.0% concentrations, CP film exhibited the highest EB values, 12.24% and 12.37%, respectively.

#### Transparency and optical properties

**Fig. 5:**
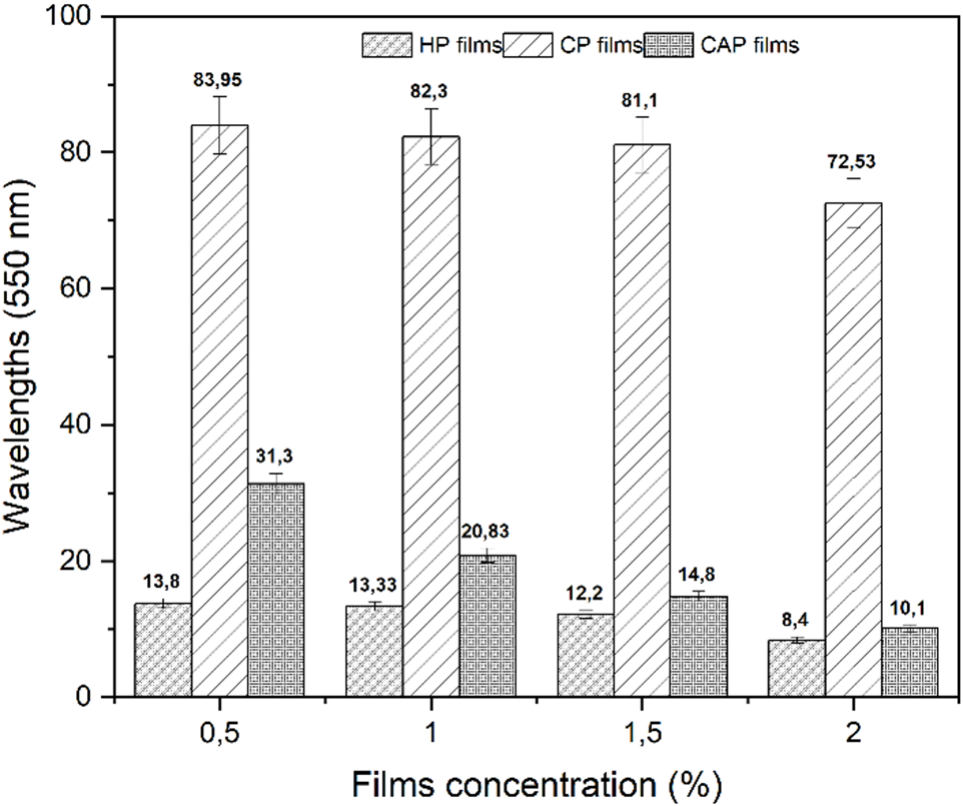
Transparency of biodegradable films at different concentrations. Different bars indicate significant differences (one-way ANOVA followed by Tukey’s LSD test, SPSS 25.0, *p* < 0.05).

The transparency of packaging material greatly influences their consumer approval and overall look^58^. The light absorbance of the pectin films was measured at a wavelength of 550 nm as shown in Fig. 5. The results indicate that the light absorbance of all films decreased as the concentration increased. Among the assessed films, CP films exhibited the highest transparency, followed by CAP films, whereas HP films exhibited the lowest transparency. The variations in opacities among CP, CAP, and HP films can be related to differences in their structural and physicochemical characteristics. The film’s barrier ability for visible light is affected by groove surfaces and laminar structure^59^. A rougher film surface and inhomogeneous microstructures tend to increase light dispersion; consequently, the greater roughness reduces the film transparency^60^.

Table 2 presents the color parameters for the different pectin films, revealing significant differences in (L) lightness, (a) red-green, and (b) yellow-blue values across all films at various concentrations. As the concentration increased, the brightness of the films (L) decreased, with CP films exhibiting the highest value. At a 2% concentration, CAP films were brighter than HP (45.17 vs. 60.15), but HP was the opposite. For CP films, the (a) parameter exhibited negative values ranging from − 0.957 to -0.793, indicating a rather verdant color. Positive values, indicating a reddish tint, were observed in HP and CAP films, with HP films exhibiting the highest values (10.20 to 26.30), which can be attributed to several chemical factors related to the extraction medium. The high extraction temperature (90 °C) combined with the strong mineral acid may have accelerated the early stages of the Maillard reaction between residual proteins and sugars, leading to specific red toned intermediate chromophores. Regarding b, CP films showed the lowest values, ranging from 7.33 to 14.41, while HP and CAP films exhibited higher values, ranging from 31.71 to 48.66. The total color difference ∆E was significantly higher for the CAP films. This shift in color parameters may be attributed to several thermal and chemical phenomena occurring at 90 °C. Specifically, the combination of high temperature and acidic conditions may have facilitated the Maillard reaction between residual nitrogenous compounds and reducing sugars within the pectin extract. A significant difference in ∆E (total color difference) was observed for all prepared pectin films, which increased with increasing concentration. CP films demonstrated the lowest ∆E values, agreeing with the results of Liu et al.^61^.

**Table 2:** Color parameters (L, a, and b) of the different pectin films.

Films Con. (%)	L			a			b		
	CAP films	HP films	PC films	CAP films	HP films	PC films	CAP films	HP films	PC films
0.5	79.91 ± 0.35^b^	67.77 ± 1.84^a^	92.71 ± 0.32^c^	3.83 ± 0.15^b^	10.20 ± 1.42^c^	-0.957 ± 0.05^a^	31.71 ± 0.25^b^	39.15 ± 1.37^c^	7.33 ± 0.25^a^
1	76.42 ± 0.89^b^	63.53 ± 2.58^a^	91.89 ± 0.40^c^	5.45 ± 0.44^b^	12.89 ± 2.68^c^	-0.803 ± 0.04^a^	39.23 ± 0.79^b^	42.75 ± 1.77^c^	11.32 ± 1.27^a^
1.5	68.29 ± 0.54^b^	56.81 ± 0.70^a^	91.39 ± 0.94^c^	10.14 ± 0.41^b^	15.79 ± 0.50^c^	-0.783 ± 0.22^a^	46.83 ± 0.58^b^	44.05 ± 0.56^b^	14.29 ± 2.77^a^
2	60.15 ± 0.60^b^	45.17 ± 0.46^a^	91.26 ± 0.26^c^	15.09 ± 0.27^b^	26.30 ± 8.24^b^	-0.793 ± 0.03^a^	48.66 ± 0.46^b^	47.40 ± 9.12^b^	14.41 ± 1.45^a^

Means ± SD (*n* = 3) within the same row followed by different superscript letters (a, b, c) differ significantly (one-way ANOVA followed by Tukey’s LSD test, SPSS 25.0, *p* < 0.05).

#### Biodegradability test

**Fig. 6:**
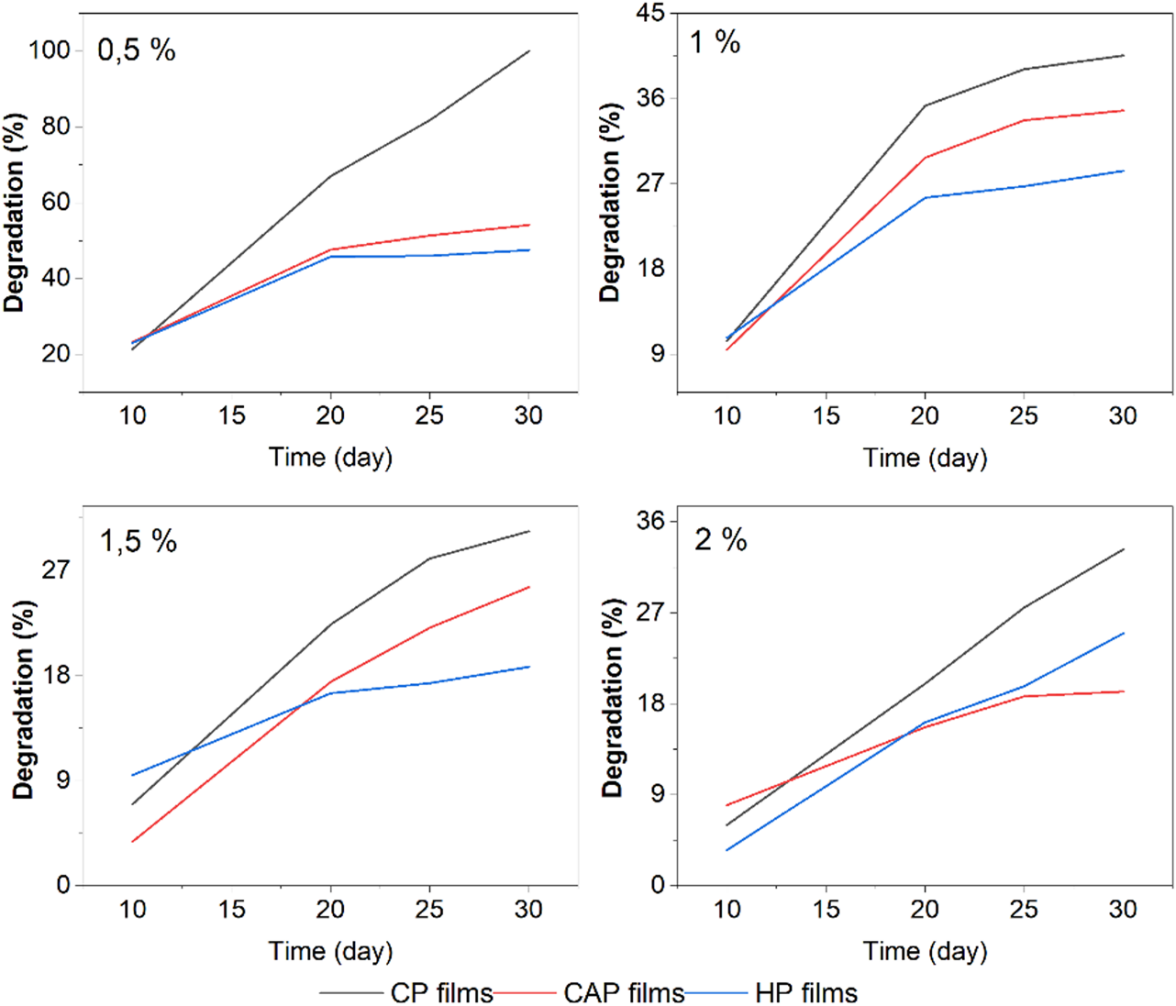
Degradation of CAP, HP, and CP edible films at different concentrations.

Biodegradability is a crucial characteristic of packaging materials^29^. Figure 6 presents the degradation results over 30 days in soil. The CP film showed a higher percentage of degradation, followed by CAP and HP. By the end of the test, the biodegradability of CP film was 51.04%, while CAP and HP films recorded 33.42% and 29.89%, respectively. It can be attributed to the extracted pectin (HP and CAP) may contain trace amounts of co extracted hemicelluloses or phenolic compounds that act as natural stabilizers, whereas commercial pectin is highly purified and lacks these secondary reinforcing elements.

As film concentration increased, the percentage of biodegradability decreased. For instance, at 0.5% concentration, the CP film exhibited the highest degradation percentage (81.75%) after 30 days, whereas lower degradation percentages of 39.09%, 28.05%, and 27.53% were observed at concentrations of 1%, 1.5%, and 2%, respectively.

Similarly, at concentrations of 0.5, 1, 1.5, and 2% of pectin, the degradation percentages of CAP films were 51.35, 33.71, 22.13, and 18.75%, respectively. For HP, the percentages of pectin films were 45.96, 26.37, 19.71, and 17.37%, respectively.

### Antioxidant properties of pectin powder and pectin edible films

**Fig. 7:**
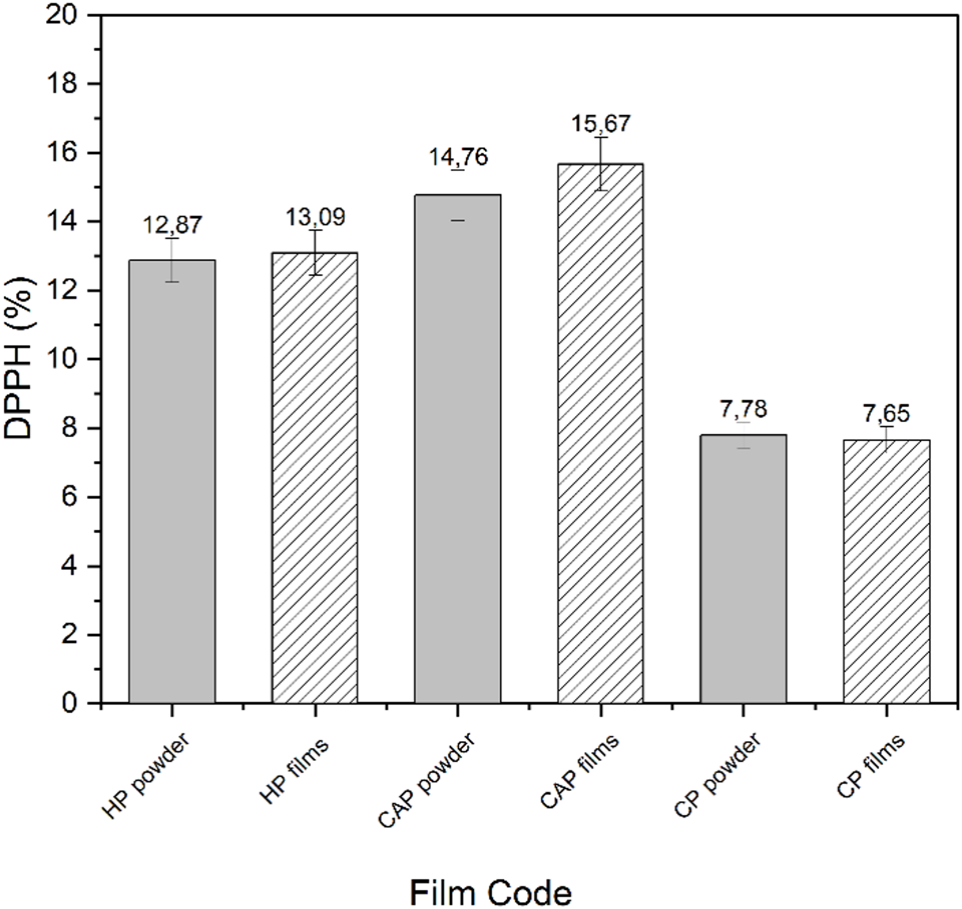
The DPPH of different pectin powder and edible films. Different bars indicate significant differences (one-way ANOVA followed by Tukey’s LSD test, SPSS 25.0, *p* < 0.05).

The antioxidant properties of pectin films and pectin powder were evaluated using the DPPH free radical scavenging assay to determine whether the extraction method using citric acid or HCl affected the antioxidant activity. As shown in Fig. 7, both the pectin films and pectin powder exhibited slight antioxidant activity, which could be attributed to the presence of some –OH groups that may scavenge free radicals, as well as the limited hydrogen-donating capacity of pectin.

CP films exhibited the lowest DPPH radical scavenging capacity, which aligns with Bello and Peresin^30^ and Yang et al.^54^. The DPPH values for pectin powder were 14.76%, 12.87%, and 7.78% for CAP, HP, and CP, respectively. The DPPH value for edible films increased compared to the pectin powder, likely due to the usage of glycerol as a plasticizer in film formation. The presence of glycerol increases the free volume within the pectin matrix, facilitating the diffusion and accessibility of naturally occurring antioxidants (such as residual phenolic compounds from the orange peel) to the DPPH radical^62^.

A notable difference was observed between the CP films and the HP and CAP films. The films made from CAP exhibited the highest DPPH value of 18.09%, followed by HP films at 15.67%, and then CP films at 9.65%. Therefore, CAP could potentially serve as a substitute for other thickening agents in products requiring oxidation stability^63^.

### Structural characterization of pectin films

#### FT-IR for different pectin films

**Fig. 8:**
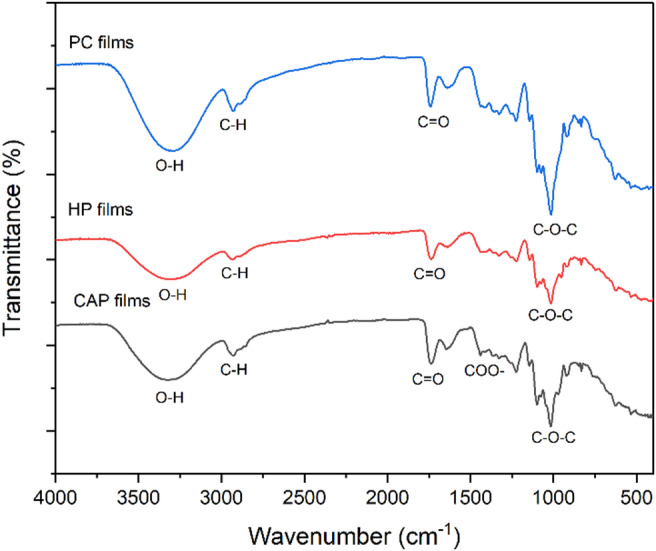
FTIR spectra of CAP, HP, and CP edible films.

The FTIR spectra of extracted pectin (CAP and HP) films and a commercial pectin film (CP) were analyzed as shown in Fig. 8, All the films exhibit identical peaks: the range of 3281–3329 cm^− 1^ corresponds to O-H bond stretching, while the peaks near 2935 cm^− 1^ are associated with C–H stretching of methane groups inside polymer chains and the methyl group of methyl ester. The peaks at 1739–1744 cm^− 1^ correspond to the C=O stretching vibration of esterified carboxyl groups. Carboxylate groups exhibit two peaks: the first, ranging from 1638 to 1648 cm^− 1^, and the second, a lesser peak between 1410 and 1440 cm^− 1^, which corresponds to the symmetric stretching vibration of COO-^52^. The peaks between 1144 cm^− 1^ and 1014 cm^− 1^ were referred to C–O–C stretching vibration of polymer chain structure^64^. The difference in the locations of the peaks are due to the protocol of extraction and the physical state of the samples. The difference in the location of COO is due to the galacturonic acid chain^52^. The peaks between (1000–1145 cm^− 1^) are due to glycosidic linkages between sugar units^65^. The absorption patterns between (800–1200 cm^− 1^) are classified as “fingerprint.” This region is unique to each compound, making its interpretation complex but essential for structural identification^66^.

#### SEM of different pectin films

**Fig. 9:**
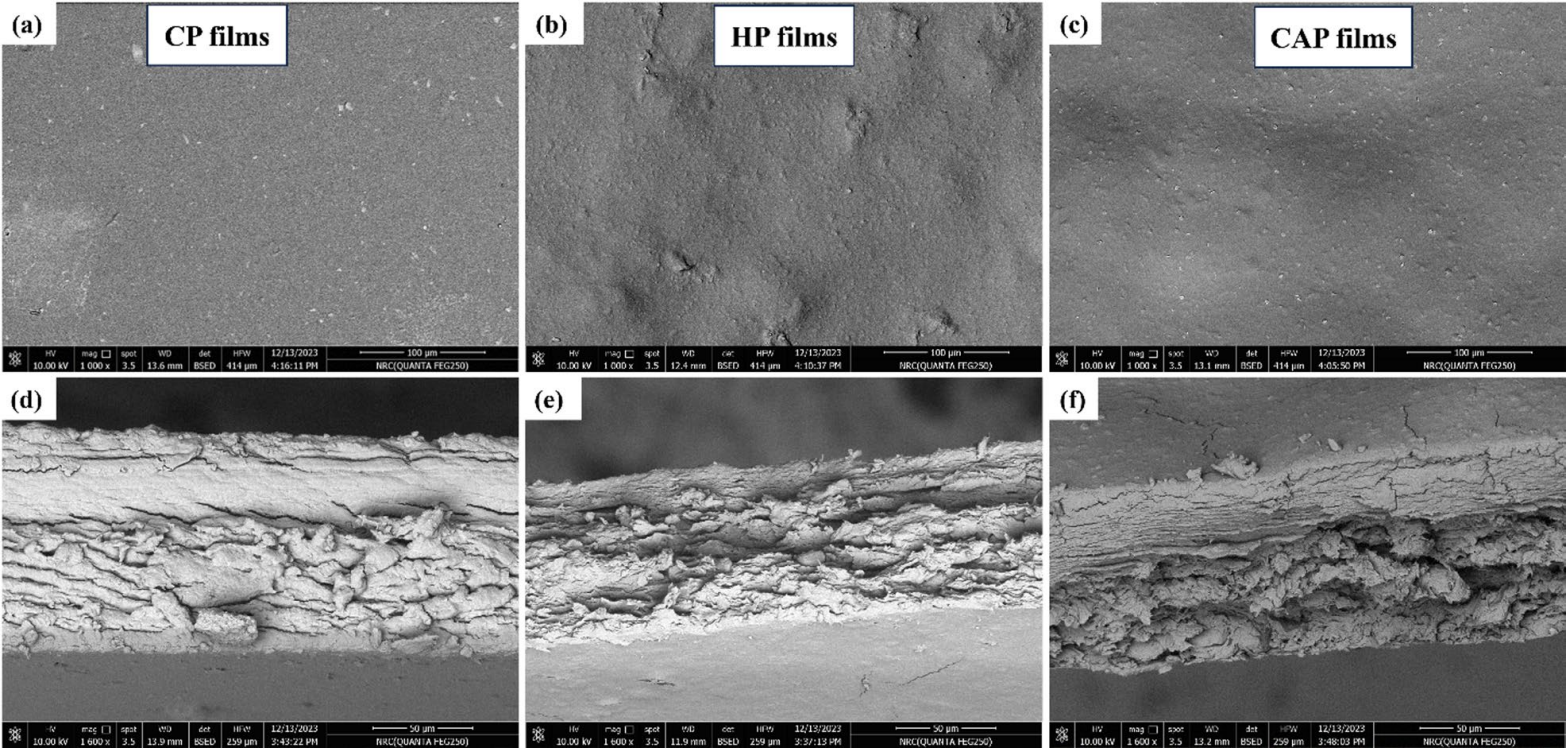
(**a**, **b**, and **c**) Surface scanning electron microscope (SEM), and (**d**, **e**, and **f**) for cross-sectional SEM images of different edible films.

Figure 9 shows SEM images of various films. The surfaces of CP film and CAP film exhibited a homogeneous structure with a smooth outside; however, minor cracks were present in the cross-section. The HP film exhibited a rough surface with a flaky morphology in its cross-section; that is due to its low tensile strength values^26,67^.

#### XRD for different pectin and biodegradable films

**Fig. 10:**
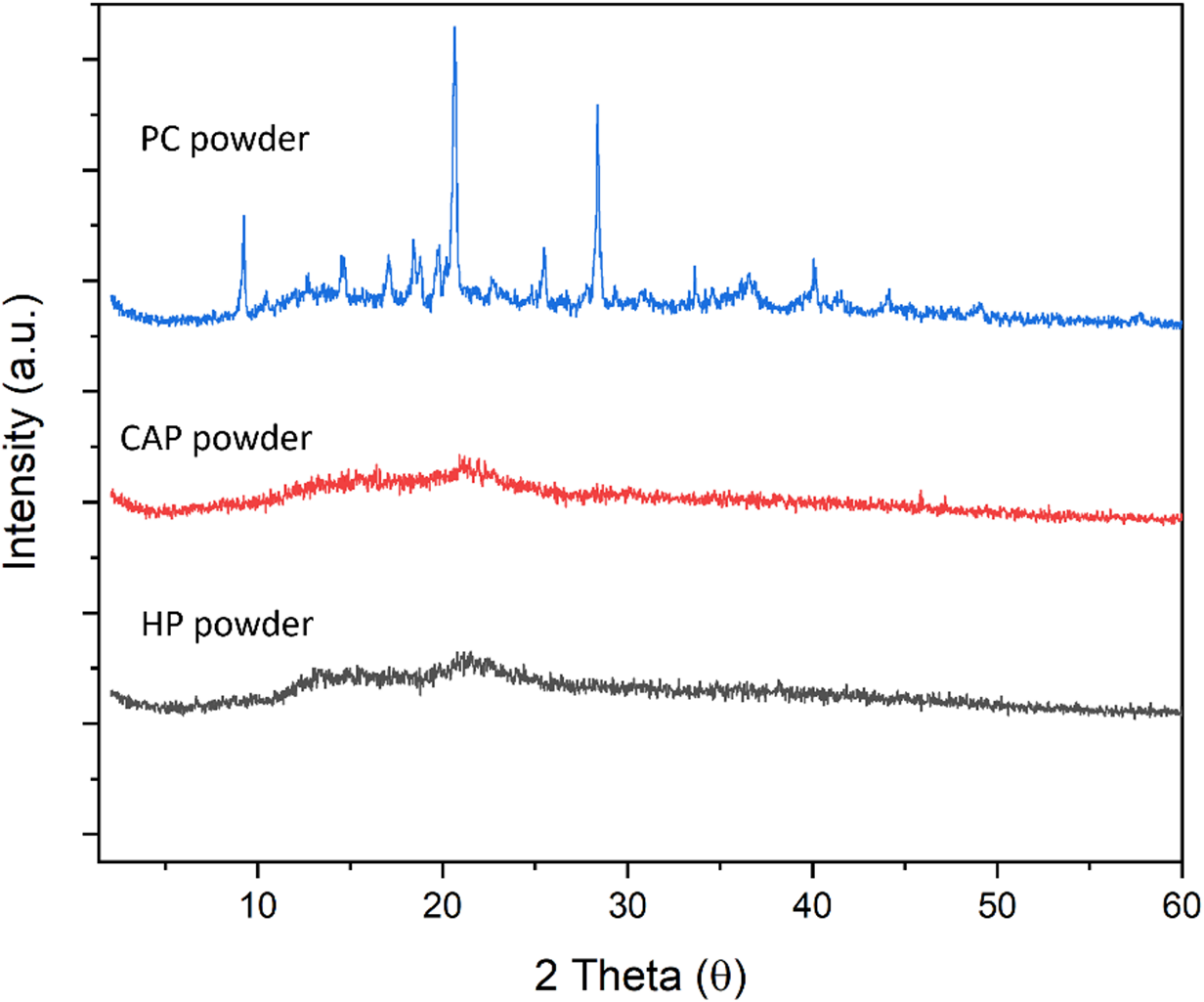
XRD patterns of pectin extracted (CAP, HP) and CP powder.

X-Ray Diffraction (XRD) examination was performed to examine the structural properties (crystalline or amorphous) of CP, CAP, and HP. A succession of pronounced peaks generally signifies crystallinity, while their absence implies an amorphous structure^68^. Figure 10 illustrates that the XRD pattern for CP exhibited a crystalline structure, characterized by strong diffraction peaks at 9.236°, 14.618°, 18.425°, 19.751°, 20.674°, 25.517°, and 28.3748° (2θ).

**Fig. 11:**
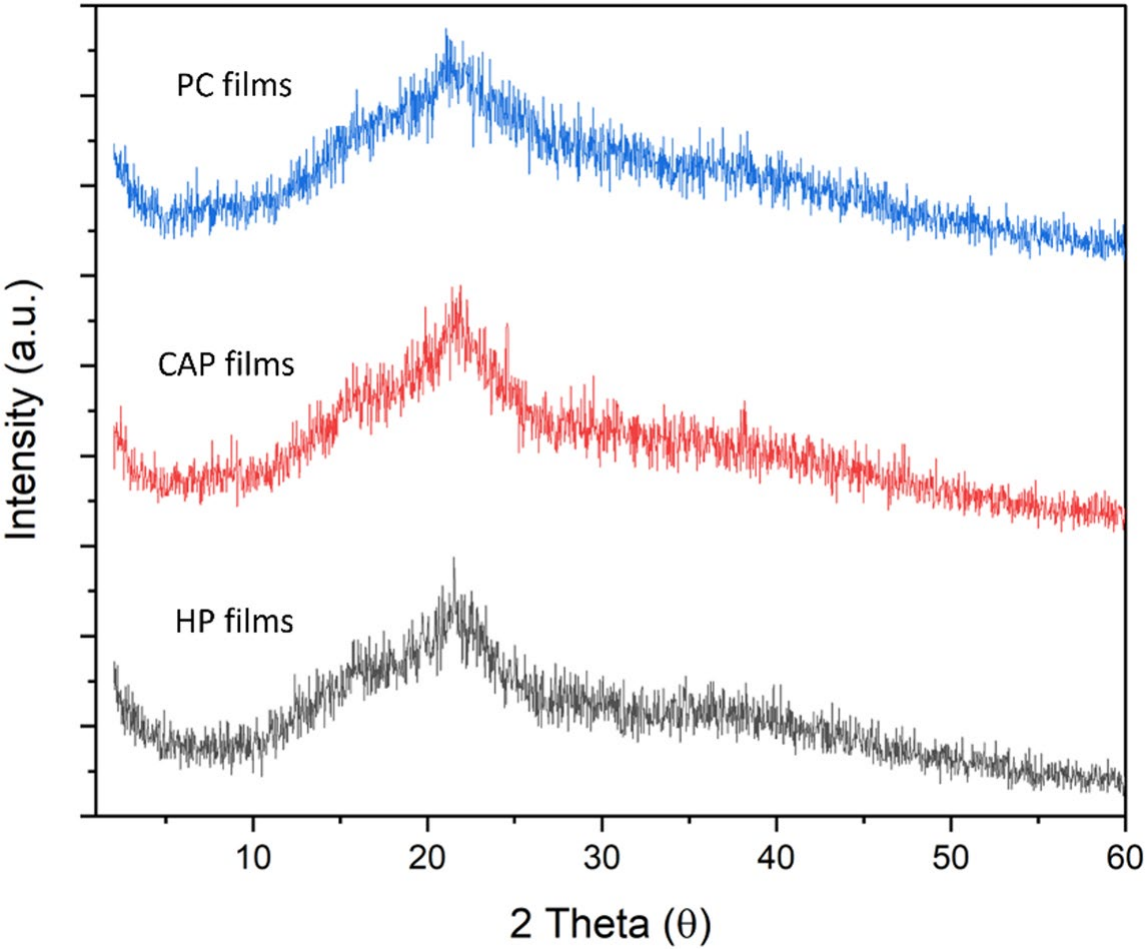
XRD patterns of CAP, HP, and CP edible films.

These findings correspond with those observed by Younis et al.^27^. Conversely, CAP indicated an amorphous structure with a peak at a 2θ value of 21.163°. Similarly, HP showed sharp peaks at 2θ values of 13.442° and 21.070°, which were consistent with the findings of Zheng et al.^68^. For the different films made from pectin extracted from citric acid CAP, HP, and CP, Fig. 11 shows their XRD patterns. In CP and HP films, the characteristic peaks (2^θ^) at 20.95° and 21.46° remained with a slight shift, but the diffraction peaks disappeared after film formation. This result indicates the strong interaction between glycerol and pectin, identical to the findings reported by Zheng et al.^68^. Meanwhile, CAP film exhibited a characteristic peak (2^θ^) at 21.64° with only a slight shift.

## Conclusions

Based on a comparison of hydrochloric acid (HCl) and citric acid extraction methods for orange peel pectin, this study concludes that citric acid is the superior and recommended choice. Although HCl extraction yielded slightly more pectin 15.15% compared with citric acid 14.44%, the minimal difference does not justify its negative environmental impact. Critically, pectin from both methods exhibited identical industrial-quality functional properties, including equivalent esterification values and commercially acceptable levels of methoxyl and anhydronic acid, making them suitable for applications like edible films.

The research emphasizes that citric acid extraction is a sustainable, biodegradable alternative that successfully transforms waste into valuable biopolymers, supporting circular economy principles. By proving that an eco-friendly method can maintain pectin quality and yield, the study provides a viable path for sustainable pectin production and the development of biodegradable packaging from agricultural waste.

## Supplementary Information

Below is the link to the electronic supplementary material.

Supplementary Material 1

## Data Availability

The datasets generated and/or analyzed of the current study are available from the corresponding author on reasonable request.
