# Supplementary material for: Synthesis and characterization of edible films using high methoxyl pectin extracted from orange peel
Source: Sci Rep. 2026 Apr 2;16:11364. doi: 10.1038/s41598-026-43924-8 (PMC13049073; doi:10.1038/s41598-026-43924-8)
Supplement: Supplementary file 1 — Supplementary Material 1 [file 41598_2026_43924_MOESM1_ESM.docx]

**Supplementary**

- - 1. **Pectin yield**

The dried pectin was milled into a fine powder and stored for analysis. The following **Eq.** **S1** was used to determine the pectin yield^1,2^.

$$\mathbf{Yield}\left( \boldsymbol{\%} \right)\mathbf{=}\frac{\mathbf{Amount of dried pectin extracted in gram}\left( \mathbf{g} \right)}{\mathbf{Amount of sample taken upfor extraction in gram}\left( \mathbf{g} \right)}\boldsymbol{\times100 (S1)}$$

- - 1. **Moisture content**

Pectin was weighed (Wt.) and subjected to drying for 3 hours at 105 °C in an oven until a stable weight (W) was attained at room temperature. The wet moisture content was ultimately determined using **Eq. S2**^3,4^.

$$\mathbf{Moisture content}\left( \boldsymbol{\%} \right)\boldsymbol{=}\frac{\boldsymbol{W}_{\boldsymbol{t}}\boldsymbol{-W}}{\boldsymbol{W}}\boldsymbol{\times100 (S}\boldsymbol{2)}$$

- - 1. **Ash content**

Approximately 2 g of the pectin sample was measured, placed into pre-weighed crucibles, and heated for 4 hours at 550°C in a muffle furnace. The specimen was then cooled in a desiccator. The following **Eq. S3** was used to determine the ash content^4,5^.

$$\mathbf{Ash content}\left( \boldsymbol{\%} \right)\boldsymbol{=}\frac{\boldsymbol{weight of ash}}{\boldsymbol{weigh of pectin}}\boldsymbol{\times100 (S}\boldsymbol{3)}$$

- - 1. **Equivalent weight and Methoxyl content**

5 ml of ethanol (95%) was added to a 250 ml conical flask containing 0.5 g of pectin. Subsequently, 1 g of NaCl and 100 mL of distilled water were added. Finally, six drops of the phenol red indicator were added. A purple color indicated the endpoint of the titration with 0.1 N NaOH, and then applying **Eq. S4** to determine the equivalent weight^3^. The methoxyl content was determined using **Eq. S5** according to the method described by Akhter, et al. ^6^.

$$\mathbf{Equivalent weight}\left( \boldsymbol{EW} \right)\boldsymbol{=}\frac{\boldsymbol{weight of sample (g)}}{\boldsymbol{ml.of alkali\times Normality of alkali}}\boldsymbol{\times1}\boldsymbol{0}\boldsymbol{00 (S}\boldsymbol{4)}$$

$$\mathbf{Methoxyl content}\left( \boldsymbol{\%} \right)\boldsymbol{=}\frac{\boldsymbol{ml.of alkali\times Normality of alkali\times3.1}}{\boldsymbol{weight of sample (g)}}\boldsymbol{(S}\boldsymbol{5)}$$

- - 1. **Total anydrouronic acid (AUA) and Degree of Esterification**

The total AUA and degree of Esterification (DE) for the pectin extract were determined using **Eqs.** **S6** and **S7** according to the method described by Diriisa, et al. ^7^

$$\boldsymbol{AUA}\left( \boldsymbol{\%} \right)\mathbf{=}\frac{\boldsymbol{176\times0.1}\boldsymbol{z}\boldsymbol{\times100}}{\boldsymbol{w}\boldsymbol{\times1000}}\mathbf{+}\frac{\boldsymbol{176\times0.1}\boldsymbol{y}\boldsymbol{\times100}}{\boldsymbol{w}\boldsymbol{\times1000}}\mathbf{(S6)}$$

$$\boldsymbol{DE}\left( \boldsymbol{\%} \right)\mathbf{=}\frac{\boldsymbol{176\times Meo}}{\boldsymbol{31\times AUA}}\boldsymbol{\times100 (S7)}$$

**Where Z** represents the volume of NaOH obtained from the **EW** assessment (ml), **y** is the volume of NaOH derived from the methoxyl content analysis (ml), and **w** indicates the mass of the sample (g).


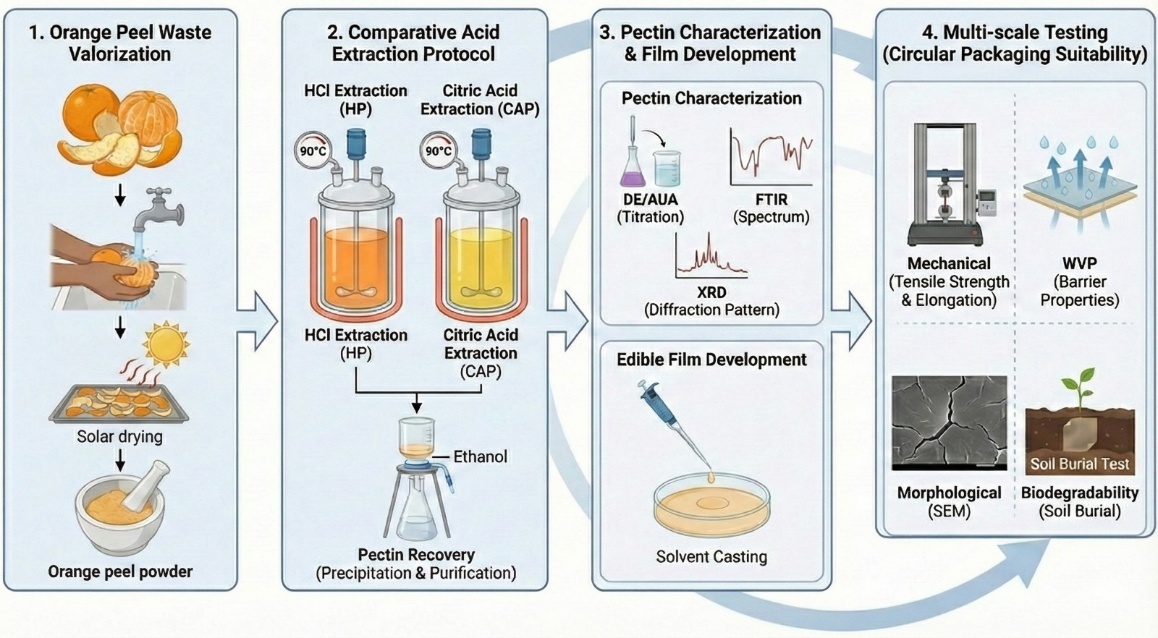


**Figure S1: Schematic of pectin extraction through HCl/CAP, pectin characterization, edible film Synthesis, and circular packaging testing (Image was created using Google's Nano Banana pro (2025)).**

**References**

1 Dong, H. *et al.* Physicochemical properties of pectin extracted from navel orange peel dried by vacuum microwave. *LWT* **151**, 112100, doi:<https://doi.org/10.1016/j.lwt.2021.112100> (2021).

2 Rodsamran, P. & Sothornvit, R. Preparation and characterization of pectin fraction from pineapple peel as a natural plasticizer and material for biopolymer film. *Food and Bioproducts Processing* **118**, 198-206, doi:<https://doi.org/10.1016/j.fbp.2019.09.010> (2019).

3 Lekhuleni, I. L. G., Kgatla, T. E., Mashau, M. E. & Jideani, A. I. O. Physicochemical properties of South African prickly pear fruit and peel: Extraction and characterisation of pectin from the peel. **6**, 178-191, doi:doi:10.1515/opag-2021-0216 (2021).

4 Ghoshal, G. & Negi, P. Isolation of pectin from kinnow peels and its characterization. *Food and Bioproducts Processing* **124**, 342-353, doi:<https://doi.org/10.1016/j.fbp.2020.09.008> (2020).

5 Maskey, B., Dhakal, D., Pradhananga, M. & Shrestha, N. K. Extraction and process optimization of bael fruit pectin. *Food Sci Nutr* **6**, 1927-1932, doi:10.1002/fsn3.761 (2018).

6 Akhter, M. J., Sarkar, S., Sharmin, T. & Mondal, S. C. Extraction of pectin from powdered citrus peels using various acids: An analysis contrasting orange with lime. *Applied Food Research* **4**, 100614, doi:<https://doi.org/10.1016/j.afres.2024.100614> (2024).

7 Diriisa, M., Gafuma, S., Kyosaba, P. & Richard, N. Characterization of Pectin from Pulp and Peel of Ugandan Cooking Bananas at Different Stages of Ripening. *Journal of Food Research* **9**, 67-67, doi:10.5539/jfr.v9n5p67 (2020).
